# Home confinement during the COVID-19: day-to-day associations of sleep quality with rumination, psychotic-like experiences, and somatic symptoms

**DOI:** 10.1093/sleep/zsab029

**Published:** 2021-02-10

**Authors:** Péter Simor, Bertalan Polner, Noémi Báthori, Rebeca Sifuentes-Ortega, Anke Van Roy, Ariadna Albajara Sáenz, Alba Luque González, Oumaima Benkirane, Tamás Nagy, Philippe Peigneux

**Affiliations:** Institute of Psychology, Eötvös Loránd University, Budapest, Hungary; UR2NF, Neuropsychology and Functional Neuroimaging Research Unit at CRCN – Center for Research in Cognition and Neurosciences and UNI – ULB Neurosciences Institute, Université Libre de Bruxelles, Brussels, Belgium; Department of Cognitive Science, Budapest University of Technology and Economics, Budapest, Hungary; Department of Cognitive Science, Budapest University of Technology and Economics, Budapest, Hungary; UR2NF, Neuropsychology and Functional Neuroimaging Research Unit at CRCN – Center for Research in Cognition and Neurosciences and UNI – ULB Neurosciences Institute, Université Libre de Bruxelles, Brussels, Belgium; UR2NF, Neuropsychology and Functional Neuroimaging Research Unit at CRCN – Center for Research in Cognition and Neurosciences and UNI – ULB Neurosciences Institute, Université Libre de Bruxelles, Brussels, Belgium; UR2NF, Neuropsychology and Functional Neuroimaging Research Unit at CRCN – Center for Research in Cognition and Neurosciences and UNI – ULB Neurosciences Institute, Université Libre de Bruxelles, Brussels, Belgium; Department of Biological and Health Psychology, Universidad Autónoma de Madrid, Madrid, Spain; UR2NF, Neuropsychology and Functional Neuroimaging Research Unit at CRCN – Center for Research in Cognition and Neurosciences and UNI – ULB Neurosciences Institute, Université Libre de Bruxelles, Brussels, Belgium; Institute of Psychology, Eötvös Loránd University, Budapest, Hungary; UR2NF, Neuropsychology and Functional Neuroimaging Research Unit at CRCN – Center for Research in Cognition and Neurosciences and UNI – ULB Neurosciences Institute, Université Libre de Bruxelles, Brussels, Belgium

**Keywords:** coronavirus, sleep, rumination, somatic symptoms, psychotic-like, tress

## Abstract

Due to the coronavirus disease 2019 (COVID-19) pandemic, populations from many countries have been confined at home for extended periods of time in stressful environmental and media conditions. Cross-sectional studies already evidence deleterious psychological consequences, with poor sleep as a risk factor for impaired mental health. However, limitations of cross-sectional assessments are response bias tendencies and the inability to track daily fluctuations in specific subjective experiences in extended confinement conditions. In a prospective study conducted across three European countries, we queried participants (*N* = 166) twice a day through an online interface about their sleep quality and their negative psychological experiences for two consecutive weeks. The focus was set on between- and within-person associations of subjective sleep quality with daytime experiences, such as rumination, psychotic-like experiences, and somatic complaints about the typical symptoms of the coronavirus. The results show that daily reports of country-specific COVID-19 deaths predicted increased negative mood, psychotic-like experiences, and somatic complaints during the same day and decreased subjective sleep quality the following night. Disrupted sleep was globally associated with negative psychological outcomes during the study period, and a relatively poorer night of sleep predicted increased rumination, psychotic-like experiences, and somatic complaints the following day. This temporal association was not paralleled by daytime mental complaints predicting relatively poorer sleep quality on the following night. Our findings show that night-to-night changes in sleep quality predict how individuals cope the next day with daily challenges induced by home confinement.

**Statement of Significance**

We examined whether night-to-night changes in sleep quality predict changes next day psychological experiences and whether daytime psychological experiences predict changes in sleep quality the following night during home confinement due to the coronavirus disease 2019 (COVID-19) pandemic. Subjective sleep was associated with worse outcomes in general and predicted increased rumination, psychotic-like experiences, and somatic complaints about the symptoms of the coronavirus the following day. Nevertheless, daytime negative psychological experiences were not predictive of poorer sleep quality the following night. Daily reports of negative psychological experiences were not independent of the perceived context of the pandemic: country-specific COVID-19 deaths were associated with increased negative mood, psychotic-like experiences, and somatic complaints during the same daytime and decreased subjective sleep quality the following night.

## Introduction

In December 2019, a new coronavirus severe acute respiratory syndrome coronavirus 2 (SARS-CoV-2) was identified as the pathogen of an acute respiratory syndrome reported first in Wuhan, China [1]. The World Health Organization announced the disease (eventually named coronavirus disease 2019 [COVID-19]) as a public health emergency of international concern [2], and, in the next 3 months, the COVID-19 continued to spread all over the world rapidly growing into a global pandemic. In order to slow the propagation of the virus as well as to attenuate the impact on healthcare systems, many of the countries introduced unprecedented measures of home confinement requiring individuals to stay at home and limit outdoor activities to the most necessary purposes. The restrictions drastically changed many of the individuals’ daily routines that within the menacing context of the pandemic could lead to severe mental health complaints [3, 4], requiring health professionals to consider the psychological impact of COVID-19 [5,6].

A number of cross-sectional surveys conducted amongst front-line medical staff [7, 8], university students [9, 10], and the general population [11–13] corroborated the concerns regarding mental health by reporting a pronounced increase in anxiety, depression, and symptoms of posttraumatic stress disorder (PTSD). Beyond these warning signs of psychological distress, a great proportion of the respondents reported frequent sleep difficulties [11, 13, 14] and poor sleep emerged as an important risk factor for mental health complaints [3]. Additionally, disrupted sleep mediated the link between threat perception (measured by the COVID-19 death count) and negative emotions in a longitudinal study [15], and those who reported more sleep difficulties during than before the confinement exhibited higher levels of depression, anxiety, and stress [16]. These findings are in line with previous studies showing the critical impact of poor sleep on the development and maintenance of mental health complaints [17–20] and the putative role of healthy sleep in emotional adaptation [21–24]. During the confinement, reduced physical activity and lower exposure to daylight, irregular sleep-wake schedules, difficulties following good sleep habits, excessive and anxiety-provoking media use, increased levels of stress, and social isolation may have a deleterious impact on sleep quality and render individuals more vulnerable to mental health problems [25]. Therefore, monitoring sleep complaints during the pandemic is particularly relevant from a mental health perspective.

Limitations of the above studies are that sleep quality and daytime symptoms were measured retrospectively (e.g. asking participants to rate sleep quality during the previous weeks or month) and data were collected using cross-sectional designs. Although the approach allows quick and economical assessments in large samples, it is extremely difficult in such designs to address the directionality of the associations (i.e. whether sleep disruption temporally predicts daytime dysfunctions or the other way around). In addition, retrospective questionnaires might be more prone to biases [26, 27], especially when individuals are queried in extremely unusual and stressful circumstances such as the COVID-19 pandemic. Studies indicate that retrospective self-reports are strongly biased by negative mood states [27], are subject to contamination between scales due to response bias tendencies [28], and show little agreement with prospectively assessed measures of similar variables [29]. Furthermore, retrospective cross-sectional studies can only account for differences between individuals and neglect intraindividual variability (i.e. moment-to-moment changes in sleep and mental complaints within the same individuals), whereas prospective assessments point to considerable within-participant variability in sleep quality [30, 31], daytime affective states [32, 33], and even in states associated with personality traits that are considered to be stable over time [34].

To overcome these limitations, we conducted a 2-week, prospective study investigating the associations between subjective sleep quality and daytime experiences during the confinement of the COVID-19 pandemic. Our prospective data collection allowed us to simultaneously examine associations across and within individuals. Hence, we tested whether individuals with poor sleep exhibited a higher level of mental health complaints during the 2-week assessment period, and whether subtle night-to-night fluctuations in sleep quality were prognostic of increased negative experiences the next day (within individuals). To address the temporal directionality of these associations, we examined whether subtle changes in daytime reports predicted changes in subjective sleep quality the following night.

Widely used measures of anxiety, depression, and stress provide efficient means to estimate the severity of negative affect and the prevalence of psychopathological conditions [3, 12] but are not capable of capturing more specific and transient mental experiences that may impose an emotional impact on individuals during home confinement. Here, we focused on three factors particularly relevant within the context of the COVID-19 pandemic: rumination, psychotic-like experiences, and somatic complaints mimicking the symptoms of COVID-19. Rumination refers to repetitive, intrusive, and hardly controllable thoughts about self-relevant situations and their underlying causes and appears as a transdiagnostic factor in various psychopathological states [35, 36]. Rumination exhibits transient, state-like variations in healthy individuals and is associated with neurophysiological indices of impaired emotional adaptation [37]. We assumed that the unpredictable nature of the pandemic might facilitate ruminative thoughts about the virus and the confinement, and, given the previously reported links between rumination and sleep quality [38], we expected that increased rumination would be linked to impaired sleep. Importantly, rumination predicts key mental health outcomes such as depression [39] and paranoia [40].

Psychotic-like experiences are unusual subjective experiences that phenomenologically resemble the symptoms of psychosis at a subclinical level (e.g. difficulties in controlling thoughts, cognitive anomalies, strange perceptions, and paranoid ideas), which are relatively common in the general population [41]. Psychotic-like experiences also exhibit considerable moment-to-moment fluctuations [42, 43] and may increase under stressful circumstances [42, 44] and after disrupted sleep [30, 43, 45]. We reasoned that psychotic-like experiences represented another particularly relevant psychological reaction to the stressful aspects of the confinement periods such as social isolation [46] and increased uncertainty of the environment. From a public mental health perspective, it is crucial that distressing subthreshold psychotic-like experiences predict the risk of developing clinically relevant psychosis [47]. Coronavirus anxiety (the fear of obtaining the virus) emerged as a novel mental health issue of severe concern [48] that was associated with a number of psychological difficulties. Therefore, somatic complaints mimicking the most common symptoms of COVID-19 were examined as a measure of somatic symptom severity [49] possibly related to the health anxiety provoked by the pandemic. Although day-to-day (intra-individual) fluctuations of somatic symptoms were mainly evidenced in clinical populations [50, 51], healthy individuals might also express pronounced variability in somatic symptoms in the context of the unprecedented coronavirus pandemic. We thus hypothesized that sleep disruptions would be associated with rumination, psychotic-like experiences, and somatic complaints during the study period, and that nights of poor sleep quality would eventually lead to more symptoms the following day within an individual. Likewise, we examined the inverse direction; that is, whether daytime rumination, psychotic-like experiences, and somatization could predict worse sleep quality the following night. Additionally, we reasoned that trait-like characteristics such as proneness for a dysregulated stress response after exposure to a stressor (as reflected by PTSD-like symptoms [52, 53]) and difficulties in controlling thoughts and attention (as reflected by cognitive disorganization [54, 55]) would be predictive of increased maladaptation during the pandemic. Therefore, we examined the association of these retrospectively assessed psychopathological indices with daytime complaints over and above the hypothesized association with sleep.

## Methods

### Participants

Individuals (*N* = 728, 556 [76%] females, age = 18-69 years, Mean_age_ = 28.5, SD_age_ = 10.09) willing to participate in our study were selected from three European countries in which restrictive confinement measures were adopted. The majority of the respondents were young, university students, and their family members. Participants were contacted by advertisements placed on social media and through the web pages and mailing services of the participating universities (Université Libre de Bruxelles, Belgium; Eötvös Loránd University, Hungary; and Autonomous University of Madrid, Spain). In the first phase of the study, participants were asked to complete an online questionnaire including self-report scales of standardized questionnaires measuring sleep quality [56], depression [57], schizotypy [58], and symptoms of PTSD [59]. Demographic variables and items related to events (e.g. tested positive for COVID-19) and experiences related to the confinement (e.g. household and school- and work-related stress) were also assessed. This cross-sectional questionnaire served us to screen and select the participants for the second, prospective phase of the study. Individuals with current or prior history of neurological, psychiatric, or chronic somatic diseases; scoring above 12 on the short form of the Beck Depression Inventory (BDI-13^57^); or taking medication (except contraceptives) were excluded. In addition, we excluded participants who reported to be previously diagnosed with COVID-19 or believed that they currently suffered or had suffered but recovered from the virus. Individuals fulfilling the inclusion criteria and willing to continue participation (*N* = 246) were selected for the prospective study phase. In total, 184 individuals (146 [79%] females, age 18-69 years, M_age_ = 26.28, SD = 7.42) were assigned for the second phase of the study assessing daily questionnaires during 2 weeks. Informed consents were obtained, and the study was approved by the corresponding local ethical committees of the three participating universities: the local Ethics Committee of the Université Libre de Bruxelles, the Ethics Committee of the Eötvös Loránd University, and the Ethics Committee of the Autonomous University of Madrid, respectively.

### Procedure

**Figure 1.**
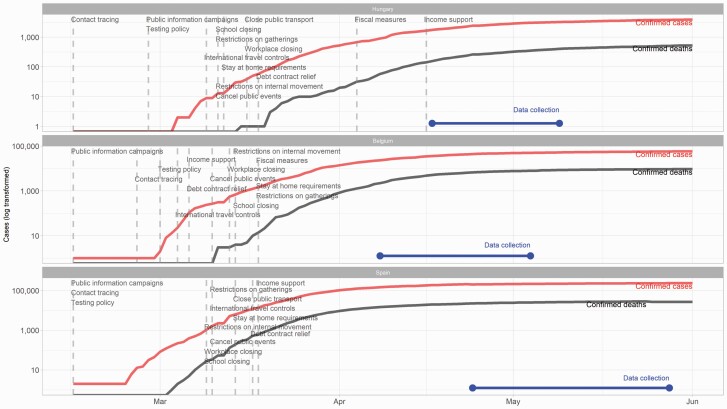
The plot shows the progress—confirmed cases and deaths—of the COVID-19 pandemic in three European countries. Dashed lines show the introduction of specific countermeasures. The blue line segment shows when the data collection took place. Data from: Hale et al. [60].

The study consisted of three phases: (1) the administration of the cross-sectional questionnaire to screen and enroll potential participants, (2) the prospective study phase asking participants to complete short questionnaires twice a day during two consecutive weeks, and (3) the debriefing phase. The same questionnaire-batteries were used for the Belgian, Hungarian, and Spanish samples using the standardized or available versions in English, Hungarian, and Spanish, respectively. Single items were created in each language by the respective native speaker authors of the research team. Items that were not available in one language were translated by the native speaker members of the research team. Research assistants invited the selected participants to sign up for the prospective phase of the study through a dedicated website where only the invited participants could register and approve their registration via a two-step authentication process. Participants signed up between April 9, 2020, and May 14, 2020, for the 2-week assessment period (see Figure 1 showing the 2-week assessment period within the timeline of the pandemic). Daily questionnaires requiring approximately 3–5 minutes per day were sent to participants via the online interface. A morning questionnaire was available between 05:00 am and 12:30 pm, and an evening questionnaire was accessible between 06:00 pm and 03:00 am. Participants were instructed to complete the morning questionnaire upon awakening and to complete the second questionnaire always before going to sleep. A reminder email of the morning questionnaire was sent between 07:00 and 09:00 am, and a reminder of the second questionnaire was sent between 07:00 and 08:00 pm. The 2-week-long prospective study period started with the first morning questionnaire the day after the participant completed the registration and ended on the 14th day with the last evening questionnaire. Morning questionnaires assessed subjective sleep quality and sleep schedules (bedtime, sleep latency, and wake-up time). The evening questionnaires consisted of items measuring mood, rumination, psychotic-like experiences, and somatic complaints comprising the most typical symptoms of the COVID-19. Participants were also asked to report daily caffeine intake and alcohol consumption and their overall satisfaction with daily activities (work, physical activity, social interactions, media use, etc.). After finishing the last questionnaire, participants were instructed to access the debriefing that consisted of a final phase providing the possibility for the participants to ask questions regarding the study and to report any issue that they considered important. Moreover, they were asked to report if they had been tested positive for COVID-19 and if they assumed that they had contracted the virus during the study period.

### Cross-sectional measures

The PTSD Checklist from the DSM-5 (The Diagnostic and Statistical Manual of Mental Disorders, Fifth Edition) (PCL-5) was used to assess PTSD-like symptoms [59]. This 20-item self-report questionnaire that assesses DSM-5 symptoms of PTSD is not only suitable to screen individuals for PTSD, but it can also be applied from a dimensional point of view to quantify the severity of symptoms of stress in clinical and subclinical populations [61]. Symptoms of depression were assessed with the short form of the Beck Depression Inventory (BDI-13). The questionnaire is suitable to screen individuals with mild, moderate, or severe symptoms of depression [57]. Sleep disturbances were measured by the respective subscale of the Pittsburgh Sleep Quality Index (PSQI [56]), a questionnaire used widely to evaluate subjective sleep quality. The sleep disturbances subscale focuses on subjective sleep complaints, such as having difficulties falling asleep, nocturnal awakenings, pain and discomfort during bedtime, as well as daytime sleepiness and fatigue. We used the Cognitive Disorganization scale of the Oxford-Liverpool Inventory of Feelings and Experiences (O-LIFE) to evaluate the cognitive aspects of trait-like schizotypy [58]. The 11 yes/no items of the scale assess difficulties in attention, concentration, decision-making, and social functioning. Validated versions of the PCL-5, BDI-13, PSQI, and O-LIFE questionnaires were available in all three languages. The levels of school- and/or work-related stress experienced as a consequence of the confinement were assessed by 7-point Likert-scales (e.g. 0 = I experience much less stress since the introduction of the confinement, 6 = I experience much more stress…).

### Morning questionnaire

Participants completed the Groningen Sleep Quality Scale (GSQS) [62] each morning. The questionnaire consists of 14 binary items that measure the extent of subjective sleep fragmentation. Previous studies indicate that the questionnaire is an adequate tool for assessing subjective sleep disruption [63]. Additionally, participants were asked to report their bedtime, sleep latency (the time they needed to fall asleep), and wake-up time. These responses were used to compute subjective sleep duration (i.e. the time elapsed between sleep onset and morning awakening). GSQS was not available in Spanish; therefore, the English version of the GSQS was translated by one of the native Spanish speakers of the research team. Two additional native speakers checked, discussed, and finalized the translations by consensus.

### Evening questionnaire

To measure ruminative, perseverative, and intrusive thoughts, items assessing state rumination (developed by Kocsel and colleagues [37]) were adapted to the context of the confinement. Three statements (“I was not able to get certain thoughts out of my mind.”; “I kept thinking about something over and over again.”; and “ I had difficulties in suppressing my thoughts about the current situation.”) were rated on a 4-point Likert scale. Items of state rumination were not available in Spanish; therefore, these were translated by the native Spanish speaker members of the research team. Psychotic-like experiences were assessed with a scale consisting of eight items that were originally adapted from two validated instruments [42, 64]. The items (rated on an 8-point Likert scale) covered perceptual anomalies (e.g. “Familiar things have seemed strange or unusual”), cognitive disorganization (e.g. “I have found it difficult to think clearly”), and paranoid ideation (e.g. “I think people have been saying or doing things to annoy me.”). The scale showed good psychometric properties and proved to be effective for the daily assessment of psychotic-like experiences in a previous study [43]. Somatic complaints were measured by items adopted from the Patient Health Questionnaire (PHQ-15) [49]. The PHQ-15 was designed to measure the prevalence of the most common body symptoms (e.g. headache and nausea). Here, we used items that overlapped with the most typical symptoms of the coronavirus, such as stomach pain, headache, chest pain, dizziness, low energy, muscle pain, and shortness of breath, and we added an extra item concerning the experience of dry cough or sore throat. Daytime mood was assessed by a single item (“Throughout the day my mood was...”) rated on an 8-point Likert scale (0 = extremely negative: sad, negative, distressed; 7 = extremely positive: happy, joyful, relaxed).

### Statistical analyses

In order to account for the nested structure of the data (repeated measures within participants), linear mixed models were fitted using the lme4 package [65] in R (v3.6.3). Every model included a random intercept and random slope per participant for the within-person-centered time-varying predictor. In case of convergence issues, random slopes were removed, and a random intercept-only model was fitted. We disentangled between- and within-person effects of time-varying variables obtained from the prospective study [66]. Within-person averages over the 2-week study period were entered to assess between-person effects (i.e. to capture differences between participants scoring high or low on a measure on average), while within-person centering was applied to examine within-person effects (i.e. to capture correlates of day-to-day deviation from the participant’s average on a measure). When we fitted models that included somatic complaints, we excluded data from eight participants who indicated, at the debriefing phase, that they believed that they had contracted the coronavirus during their participation in the study. Autocorrelation was taken into account in all the examined models detailed below. Therefore, each dependent variable was also predicted by the same variable at the preceding time point (i.e. preceding day). The reason to include an autocorrelation parameter in the models was to control for carryover effects in our measures of interest.

First, we investigated how events associated with the pandemic predicted daytime mental health complaints, sleep disruption, and sleep duration. Predictors were country-wise and worldwide numbers of COVID-related deaths for the given day (accessed from https://www.worldometers.info/coronavirus/coronavirus-death-toll/ as of May 19, 2020). In order to facilitate interpretation of coefficient estimates, the number of country-wise and global deaths per day was scaled with a division by 10 and 1000, respectively. Additionally, we calculated the number of days since the introduction of confinement in the country of the participant (Belgium: March 14, 2020; Hungary: March 28, 2020; and Spain: March 17, 2020). Time spent in confinement was expressed in weeks (number of days/7) in order to have this variable on a scale comparable to the other predictors (this was done to facilitate model fitting and interpretation of coefficients). In order to capture nonlinear effects, time spent in confinement was entered as a linear and as a quadratic term as well. Age, gender, PTSD-like symptoms (PCL-5 sum score), and cognitive disorganization were entered as person-specific time-invariant predictors. Dependent variables were the sum scores derived from the prospective measurements of rumination, psychotic-like experiences, and somatic complaints during the day, and sleep disruption and sleep duration the following night.

Then, we analyzed the relationship between sleep disruption/duration and mental health complaints on the next day. Dependent variables were the sum scores derived from the prospective measurements of rumination, psychotic-like experiences, and somatic complaints. As diagnostic plots suggested that model residuals were not normally distributed, each dependent variable was log_10_-transformed, after which model residuals did not appear to strongly violate normality. Age, gender, PTSD-like symptoms (PCL-5 sum score), cognitive disorganization, and within-person mean of subjective sleep disruption (GSQS sum score)/duration were entered as level-2 predictors, and within-person-centered subjective sleep quality/duration was entered as a level-1 predictor in the models.

In the analysis of the relationship between daytime mental health complaints and sleep disruption/duration the following night, the dependent variables were sleep quality (GSQS sum score) and sleep duration. Age, gender, PTSD-like symptoms (PCL-5 sum score), cognitive disorganization, and within-person means of mood, rumination, psychotic-like experiences, and somatic complaints were entered as level-2 predictors, and within-person-centered mood, rumination, psychotic-like experiences, and somatic complaints (of the day before sleep) were entered as level-1 predictors. The *p*-values corresponding to the analyses of sleep-related variables (both level-1 and level-2 predictors) were adjusted by the Benjamini-Hochberg procedure of false discovery rate to address the issue of multiple comparisons [67].

## Results

### Screening of participants and cross-sectional measures

**Figure 2.**
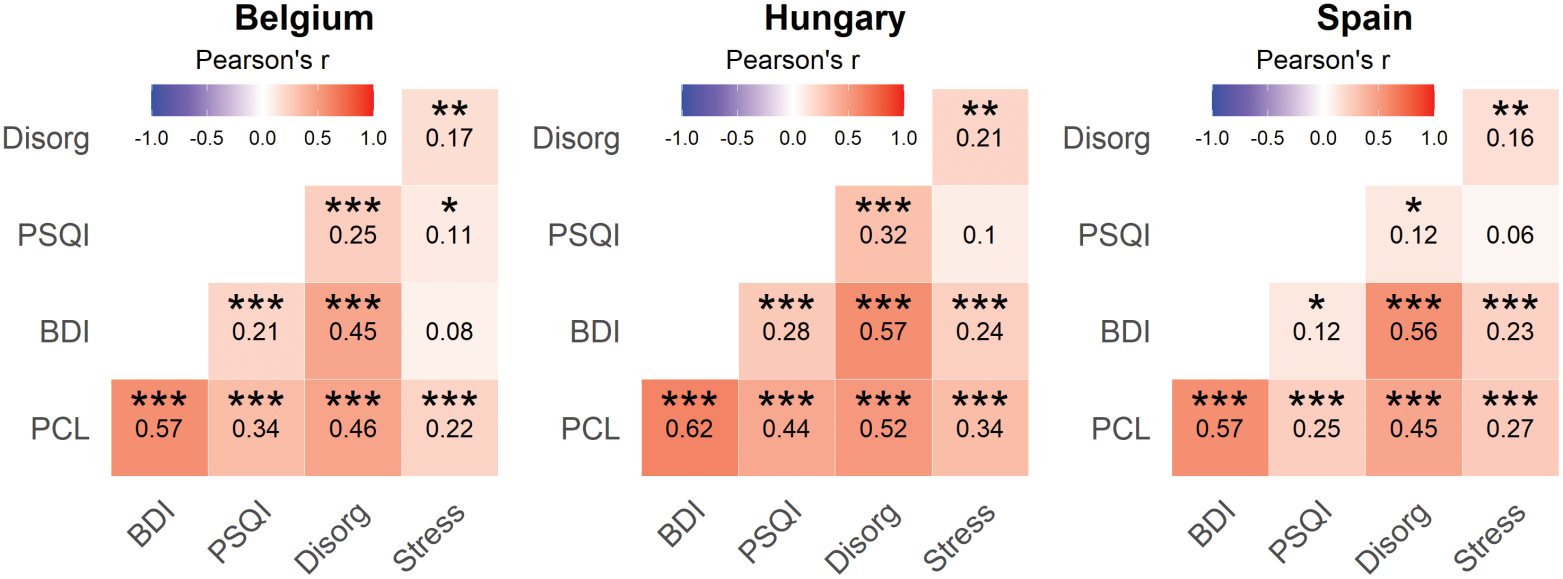
Cross-sectional survey. Pearson’s correlation coefficients between the assessed variables among the Belgian, Hungarian, and Spanish sample of respondents (*N* = 717). The colors and numbers indicate Pearson’s *r* values, and asterisks correspond to false discovery rate (FDR)-corrected *p*-values (***p < 0.001, **p < 0.01, **p < 0.05). Stress, the increase of school- and job-related stress due to the confinement; Disorg, Cognitive Disorganization; PSQI, the sleep disturbances subscale of the Pittsburgh Sleep Quality Inventory; BDI, Beck Depression Inventory; PCL, PTSD checklist.

In the first phase of the study, participants from three European countries in which restrictive confinement measures were adopted (Belgium, Hungary, and Spain) responded to a survey including items addressing cognitive disorganization, posttraumatic stress symptoms, sleep quality, depression, and general demographic data that served us to screen and select individuals for the ensuing prospective study (see Methods). Thirteen participants reported that they were tested positive for COVID-19, and their data were not considered in the subsequent analyses. The prevalence rate of clinically relevant (moderate) depression [68] in our sample (*N* = 717, 547 females) was 23% (Belgium: 25.1%, Hungary: 27%, and Spain: 18.4%), and 19.2% of the respondents (Belgium: 21.1%, Hungary: 25.6%, and Spain: 13.1%) scored over the cutoff score to identify clinically relevant symptoms of PTSD [59] corroborating previous reports about the substantial increase of mental health problems during the pandemic [3]. Although moderate differences emerged across the countries with respect to PTSD symptoms, school- and work-related stress, and cognitive disorganization (see Supplementary Materials), the psychometric measures of mental health complaints exhibited similar patterns of correlations in our subsamples (Figure 2).

### Prospective study: adherence to daily questionnaires

In total, 184 individuals (146 [79%] females, age 18-69 years, M_age_ = 26.28, SD = 7.42) were assigned for the second phase of the study assessing daily questionnaires during 2 weeks. About 22% of the respondents completed all questionnaires, and 51% completed more than 80% of the daily questionnaires. Nine percent of the participants responded to less than 50% of the morning or the evening questionnaires, and their data were excluded from further analyses yielding to the prospective data of 166 individuals. See Table 1 for a detailed description of our study sample.

**Table 1.** Descriptive statistics of the study sample

	Belgium	Spain	Hungary	Total
**Cross-sectional sample**				
Country % of cross-sectional sample (*N*)	31.2% (229)	40.5% (297)	28.2% (207)	100% (733)
Gender				
Female % (*N*)	77.7% (178)	68.0% (202)	85.0% (176)	75.9% (556)
Male % (*N*)	21.8% (50)	31.3% (93)	15.0% (31)	23.7% (174)
Education				
Less than a high school diploma % (*N*)	2.6% (6)	2.4% (7)	0.5% (1)	1.9% (14)
High school degree or equivalent % (*N*)	20.1% (46)	12.8% (38)	69.6% (144)	31.1% (228)
Bachelor’s degree % (*N*)	34.5% (79)	33.7% (100)	26.1% (54)	31.8% (233)
Master’s degree % (*N*)	34.5% (79)	36.4% (108)	1.0% (2)	25.8% (189)
Doctorate (PhD) % (*N*)	6.6% (15)	4.7% (14)	0.0% (0)	4.0% (29)
Mean age (SD)	28.2 (9.8)	32.8 (11.2)	22.8 (3.9)	28.5 (10.1)
Mean stress (SD)	19.2 (15.4)	15.9 (14.7)	22.2 (17.0)	18.7 (15.8)
Mean BDI (SD)	5.8 (5.5)	5.0 (4.6)	5.7 (5.0)	5.4 (5.0)
Mean disorganization (SD)	4.8 (3.2)	3.7 (2.8)	5.1 (3.1)	4.4 (3.1)
Mean PSQI (SD)	0.6 (0.4)	0.6 (0.4)	0.6 (0.4)	0.6 (0.4)
Exclusions				
Excluded for COVID-19 diagnosis	0.4% (1)	4.0% (12)	0.0% (0)	1.8% (13)
Chose not to participate in the prospective study % (*N*)	74.2% (170)	79.8% (237)	63.3% (131)	73.4% (538)
Excluded for low adherence % (*N*)	0.9% (2)	7.4% (22)	2.4% (5)	3.9% (29)
**Final sample (prospective phase)**				
Country % of final sample (*N*)	34.3% (57)	22.9% (38)	42.8% (71)	100.0% (166)
Gender				
Female % (*N*)	82.5% (47)	68.4% (26)	76.1% (54)	76.5% (127)
Male % (*N*)	15.8% (9)	26.3% (10)	19.7% (14)	19.9% (33)
Mean age (SD)	25.8 (6.9)	31.9 (10.1)	23.1 (4.2)	26.0 (7.6)
Mean stress (SD)	15.2 (12.5)	12.9 (9.8)	13.4 (11.3)	13.9 (11.4)
Mean BDI (SD)	3.1 (2.6)	2.6 (2.4)	2.8 (2.5)	2.9 (2.5)
Mean disorganization (SD)	4.7 (3.1)	3.0 (2.0)	4.3 (2.6)	4.1 (2.7)
Mean PSQI (SD)	0.6 (0.3)	0.4 (0.3)	0.5 (0.3)	0.5 (0.3)

The cross-sectional sample was used to select participants for the prospective, 2-week-long study.

### COVID-19-related deaths and days since the introduction of confinement predict daytime functioning

**Figure 3.**
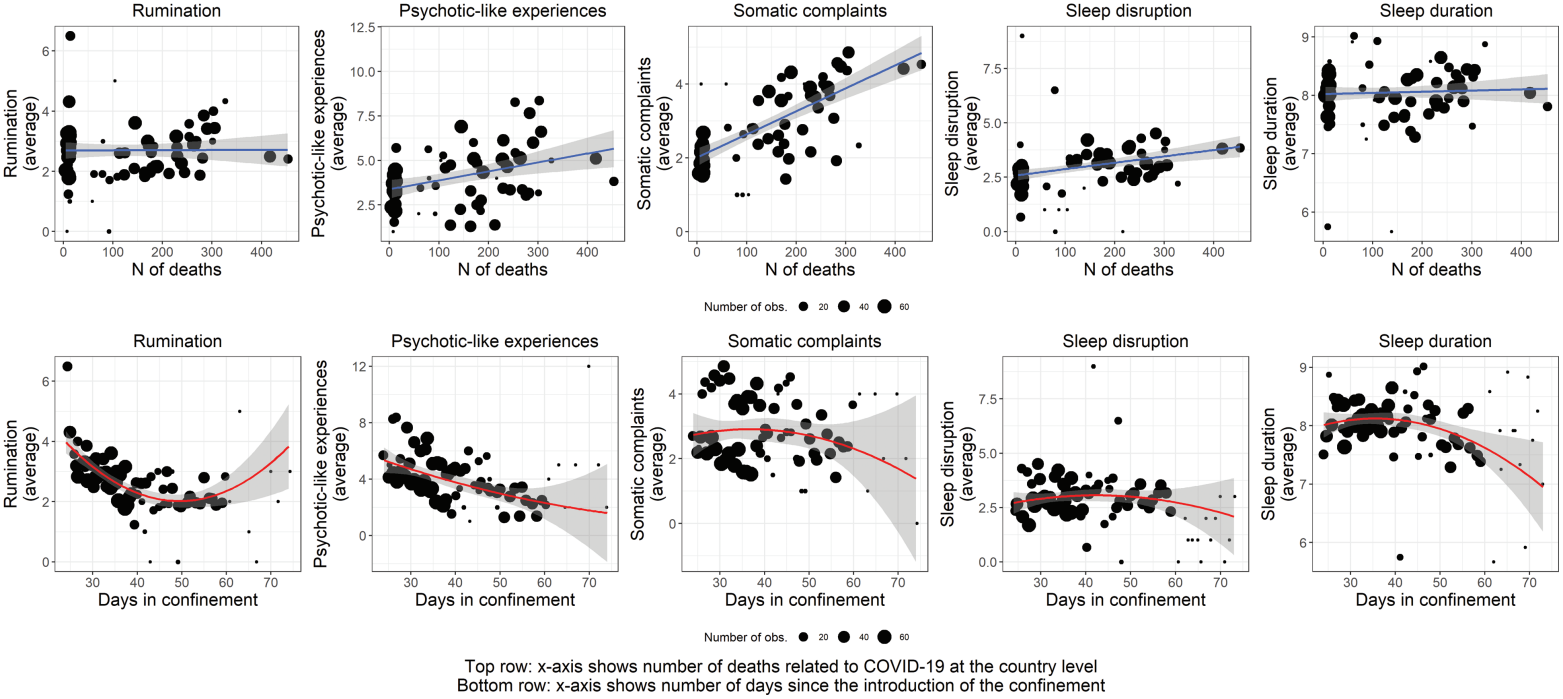
Mental health in the context of the confinement. Associations between the number of COVID-19-related deaths at the country level (linear, shown on the *x-*axis in figures in the top row) and days since the introduction of confinement (quadratic, shown on the *x-*axis in figures in the bottom row) with daytime functioning, sleep disruption, and sleep quality. Daily reports of COVID-19 deaths were predictive of increased negative mood, more psychotic-like experiences, and somatic complaints during the day and worse subjective sleep quality the following night. Daily reports of rumination, psychotic-like experiences, and somatic symptoms decreased as time elapsed since the introduction of the confinement, but we also observed a quadratic effect indicating that the amelioration of mental health exhibited a slowing trend. In the plots, each point represents the average of the given measure for a day within a country and the trend lines are weighted for the number of observations for the given day in a country. Shades show 95% CIs. Note that CIs become wider due to a lower number of observations around later time points after the introduction of the confinement.

The first analyses of the prospective study focused on the day-to-day associations of mental health complaints with the perceived context of the confinement. COVID-19-related deaths and the time spent in confinement were the objective measures reflecting the circumstances of the pandemic during the study period. We explored whether daily reports of mental health problems were associated with the daily numbers of COVID-19-related deaths worldwide and in the country of the respondents. Moreover, since the number of deaths had not been independent of the time spent since the introduction of the restrictive measures, we also examined the influence of time (expressed as a fraction of weeks) spent in confinement on daily reports of mood, rumination, psychotic-like experiences, somatic complaints, sleep disruption, and sleep duration (Table 2). The number of COVID-19-related deaths in the country predicted more psychotic-like experiences and somatic complaints during the day and worse subjective sleep quality during the following night, whereas the number of worldwide deaths predicted more somatic complaints during the day and prolonged sleep duration the following night. The number of weeks since the introduction of confinement in the country had a negative linear effect on rumination and psychotic-like experiences and a positive linear effect on sleep duration. Moreover, the number of weeks since the introduction of confinement had a positive quadratic effect on rumination and psychotic-like experiences (see Table 2 for statistical parameters). Further inspection of the plots of predicted values suggested that there was an overall improvement of mental health complaints with time spent in confinement (Figure 3). Moreover, the steepness of the curve (reflecting the gradual amelioration of daily symptoms) was decreasing with time, indicating that the amelioration of mental health complaints exhibited a slowing trend. These findings indicate that day-to-day fluctuations in mental health conditions were specifically associated with objective measures (number of deaths and days spent in confinement) reflecting the context of the confinement.

**Table 2.** The association of the number of deaths related to COVID-19 and of the days spent in confinement with mental health complaints, beyond the effects of age, gender, PTSD-like symptoms, and cognitive disorganization

Predicting daytime mental health complaints and sleep quality from COVID-19-related variables										
	Rumination		Psychotic-like experiences		Somatic complaints		Sleep disruption		Sleep duration	
Predictors	Estimates	95% CI	Estimates	95% CI	Estimates	95% CI	Estimates	95% CI	Estimates	95% CI
Intercept	0.804***	0.410 to 1.198	0.876***	0.441 to 1.312	0.395*	0.047 to 0.744	0.454*	0.103 to 0.805	7.512***	6.368 to 8.657
Age	0.004	−0.001 to 0.010	0.002	−0.005 to 0.008	−0.003	−0.008 to 0.002	0.004	−0.000 to 0.008	−0.027***	−0.041 to −0.014
Gender (male)	−0.099*	−0.185 to −0.013	−0.075	−0.182 to 0.033	−0.150***	−0.236 to −0.065	−0.083*	−0.155 to −0.012	−0.412***	−0.633 to −0.191
PTSD-like symptoms	0.008***	0.005 to 0.011	0.011***	0.007 to 0.015	0.008***	0.005 to 0.011	0.003*	0.001 to 0.006	0.004	−0.004 to 0.013
Cognitive Disorganization	0.024**	0.010 to 0.039	0.037***	0.019 to 0.054	0.029***	0.015 to 0.043	0.020**	0.008 to 0.032	−0.033	−0.070 to 0.004
***Deaths related to COVID-19* *(in country, 10/day)***	*0.001*	−*0.002 to 0.003*	***0.003*** *	***0.000 to 0.006***	***0.005*** ***	***0.003 to 0.007***	***0.003*** **	***0.001 to 0.005***	*0.003*	−*0.004 to 0.009*
***Deaths related to COVID-19 (global, 1000/ day)***	*0.004*	−*0.009 to 0.018*	*0.008*	−*0.007 to 0.022*	***0.016*** **	***0.004 to 0.028***	−*0.010*	−*0.024 to 0.004*	***0.054*** *	***0.008 to 0.100***
***Weeks spent in confinement (linear)***	−0.234***	−***0.356 to ***−***0.112***	−0.251***	−***0.383 to ***−***0.119***	−*0.080*	−*0.186 to 0.025*	−*0.056*	−*0.165to 0.053*	***0.366*** *	***0.009 to 0.723***
***Weeks spent in confinement (quadratic)***	***0.017*** **	***0.006 to 0.027***	***0.016*** **	***0.005 to 0.028***	*0.006*	−*0.004 to 0.015*	*0.003*	−*0.006 to 0.013*	−*0.030*	−*0.061 to 0.001*
Autocorrelation	0.013***	0.007 to 0.019	0.004*	0.001 to 0.008	0.002	−0.004 to 0.007	−0.004	−0.010 to 0.001	−0.063**	−0.109 to −0.017
Random effects										
σ ^2^	0.0760		0.0860		0.0526		0.0932		1.0140	
τ _00_	0.0431		0.0709		0.0420		0.0280		0.2566	
ICC	0.3618		0.4520		0.4441		0.2308		0.2019	
*N*	165		165		157		166		166	
Observations	1590		1590		1517		1928		1927	
Marginal *R*^2^/ Conditional *R*^2^	0.161/0.464		0.223/0.574		0.255/0.586		0.064/0.280		0.059/0.249	

The variables reflecting the context of the pandemic are set in italics, and significant associations between covid-related stressors and outcome measures are highlighted in bold. Daily reports of country-specific COVID-19-related deaths were associated with psychotic-like experiences and somatic complaints during the day and worse subjective sleep quality the following night. The number of global deaths predicted more somatic complaints during the day and prolonged sleep duration the following night. The time spent in confinement was inversely related to the reported levels of rumination and psychotic-like experiences and positively to sleep duration. A quadratic trend of the time spent in confinement was also observed on rumination and psychotic-like experiences, indicating that in spite of the gradual amelioration of daytime mental health complaints, prolonged time in confinement may again increase mental complaints. σ2, residual variance; τ00, variance of random intercept; τ11, variance of random slope; ρ01, correlation between random intercept and slope; ICC, intraclass correlation; *N*, number of participants. Marginal *R*^2^, variance explained by fixed effects; conditional *R*^2^, variance explained by fixed and random effects. *P*-values were computed with Satterthwaite’s approximation.

*p < 0.05; **p < 0.01; ***p < 0.001.

### Night-to-night fluctuations in sleep quality predict daytime functioning

**Figure 4.**
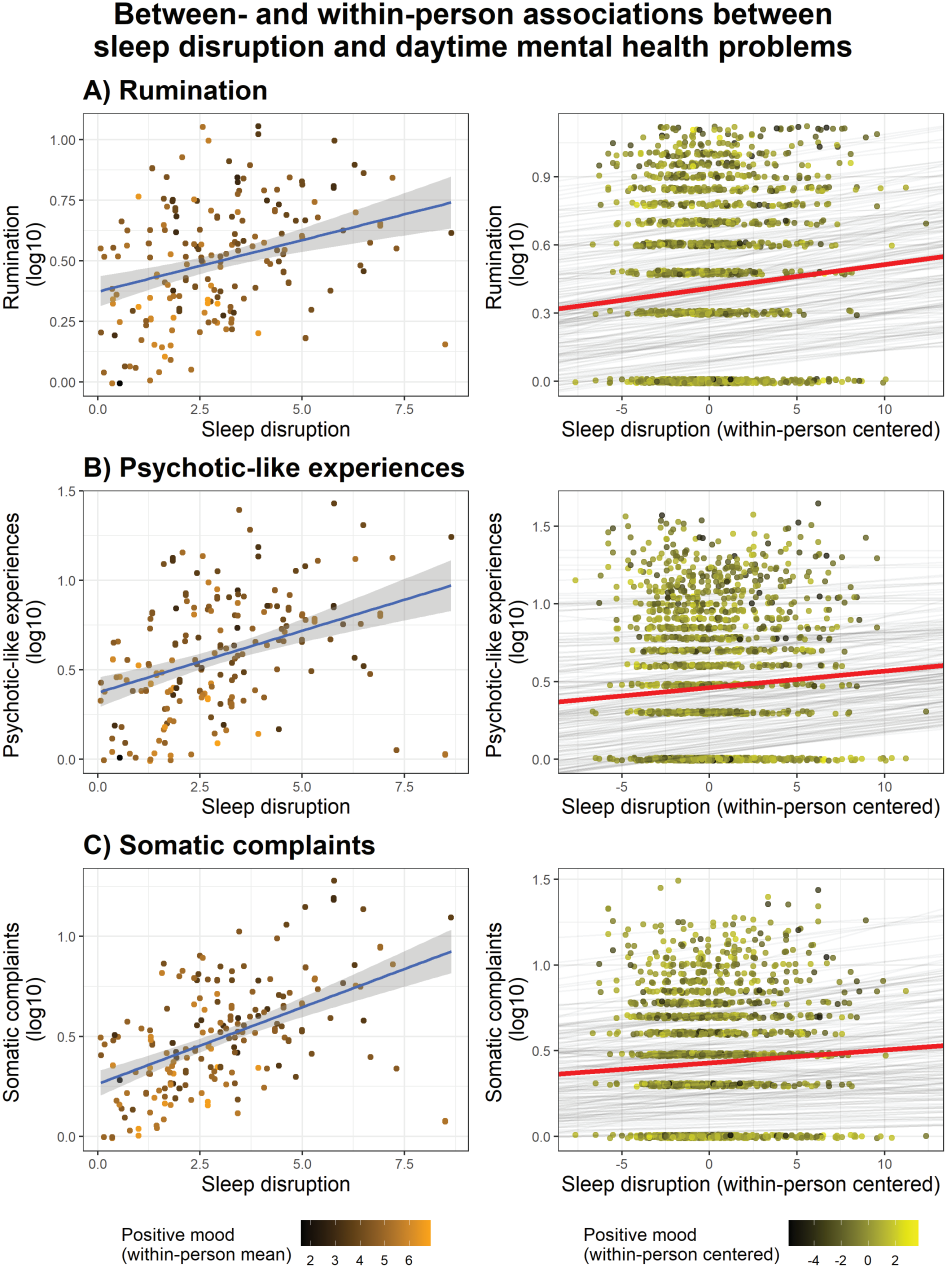
Prospective data analyses. *Left column*: Individual differences in subjective sleep disruption are associated with increased (A) rumination, (B) psychotic-like experiences, and (C) somatic complaints during the 2-week study period. The scatterplots of the left column represent the 2-week averages of sleep disruption (x-axes) and daytime experiences (y-axes). Points with brighter and darker colors indicate positive and negative ratings of mood, respectively. Note that the brighter-colored points (reflecting more positive mood) are more prominent in case of lower rumination, psychotic-like experiences, and somatic symptoms. *Right column*: Day-to-day associations between subjective sleep disruption and the next day’s rumination, psychotic-like experiences, and somatic complaints. Subjective sleep disruption as compared with the individual 2-weekly averages; that is, relatively worse nights of sleep during the study period are associated with increased (A) rumination, (B) psychotic-like experiences, and (C) somatic complaints the following day. Gray lines indicate the regression slopes of each individual fitted to their day-to-day reports of sleep quality and daytime experiences (individual means and slopes). The red lines show the regression slopes fitted to all data points (overall mean and slope). Points with brighter and darker colors indicate positive and negative ratings of mood, respectively. As indicated by the color of the data points, a more positive daily mood is associated with lower rumination, psychotic-like experiences, and somatic symptoms (see Table 2).

We examined if subjective sleep disruption (on average) was associated with increased rumination over the 2-week study period, and whether night-to-night variations in sleep quality were associated with changes in daily rumination within individuals. Figure 4A illustrates the association between sleep disruption and rumination between- and within-individuals, and statistical model parameters are specified in Table 3. Subjective sleep quality was associated with rumination in both cases: more disrupted sleep (on average) was associated with increased rumination over the 2-week study period and a relatively poorer night of sleep (i.e. reduction of sleep quality compared with the individual, 2-week average) predicted relatively increased rumination the following day. In addition, trait-like, retrospective measures of posttraumatic symptoms (assessed by the PCL-5) and cognitive disorganization were both predictive of increased rumination over the study period (see Table 3).

**Table 3.** Summary of mixed models examining associations between sleep quality and subsequent daytime functioning

Associations between sleep and mental health complaints the following day						
	Rumination		Psychotic-like experiences		Somatic complaints	
Predictors	Estimates	95% CI	Estimates	95% CI	Estimates	95% CI
Intercept	0.411	−0.156 to 0.979	0.192	−0.485 to 0.869	0.301	−0.244 to 0.847
Age	0.000	−0.005 to 0.006	−0.003	−0.010 to 0.003	−0.004	−0.009 to 0.001
Gender (male)	−0.090	−0.181 to 0.001	−0.028	−0.136 to 0.081	−0.107*	−0.195 to −0.018
PTSD-like symptoms	0.007***	0.004 to 0.010	0.009***	0.005 to 0.013	0.006***	0.003 to 0.009
Cognitive disorganization	0.020**	0.005 to 0.034	0.030**	0.012 to 0.047	0.023**	0.009 to 0.037
**Sleep disruption (within-person mean)**	0.028*	**0.006** to **0.049**	0.055***	**0.029** to **0.081**	0.057***	**0.036** to **0.078**
***Sleep disruption (within-person centered)***	***0.008*** *	***0.002*** to***0.014***	***0.011*** **	***0.005*** to***0.018***	***0.008*** **	***0.003*** to***0.013***
Sleep duration (within-person mean)	−0.034	−0.094 to 0.026	−0.009	−0.081 to 0.063	−0.012	−0.069 to 0.045
*Sleep duration (within-person centered)*	−*0.008*	−*0.024* to *0.007*	−*0.001*	−*0.018* to *0.015*	−*0.007*	−*0.020* to *0.006*
Autocorrelation (dependent variable the day before, within- person centered)	0.016***	0.010 to 0.022	0.007***	0.004 to 0.011	0.006*	0.000 to 0.012
Random effects						
σ ^2^	0.0754		0.0867		0.0539	
τ _00_	0.0416		0.0628		0.0382	
τ _11_	0.0002		0.0002			
ρ _01_	0.0438		−0.3465			
ICC	0.3644		0.4270		0.4146	
*N*	165		165		157	
Observations	1524		1524		1454	
Marginal *R*^2^/conditional *R*^2^	0.165/0.469		0.244/0.567		0.281/0.579	

The day-to-day associations between sleep quality and daytime complaints are set in italics. Uncorrected *p*-values were computed with Satterthwaite’s approximation. Correction for multiple comparisons in the case of the sleep-related predictors was addressed by the Benjamini-Hochberg procedure of false discovery rate (FDR) [66]. The significant associations between sleep and the next day’s mental health complaints surviving statistical correction (FDR) are highlighted in bold. σ2, residual variance; τ00, variance of random intercept; τ11, variance of random slope; ρ01, correlation between random intercept and slope; ICC, intraclass correlation; *N*, number of participants. Marginal *R*^2^, variance explained by fixed effects; conditional *R*^2^, variance explained by fixed and random effects.

*p < 0.05; **p < 0.01; ***p < 0.001.

Next, we performed a similar analysis regressing the same variables on psychotic-like experiences. As expected, worse sleep quality was globally associated with increased reports of psychotic-like experiences. Moreover, night-to-night changes in sleep quality within individuals were also linked to increased psychotic-like experiences the following day; that is, worse sleep quality predicted more psychotic-like experiences the next day. PTSD-like symptoms and cognitive disorganization were both predictive of increased psychotic-like experiences (see Figure 4B and Table 3).

Likewise, we investigated the links between sleep disruption and the daily experience of somatic symptoms of the COVID-19. At the between-participant level, subjective sleep disruption was positively associated with reports of somatic symptoms: the within-person average of sleep disruption was associated with a higher rate of somatic complaints over the course of the study. Regarding daily fluctuations within individuals, relatively worse sleep quality on a given night was associated with more severe somatic symptoms the following day. PCL-5 scores and cognitive disorganization both emerged as significant predictors of (increased) somatic symptoms in the statistical model (see Figure 4C and Table 3).

In sum, disrupted sleep was associated with increased rumination, psychotic-like experiences, and somatic symptom severity as measured prospectively during the 2-week study period. Moreover, night-to-night variations in subjective sleep quality predicted next day’s functioning, indicating that a relatively worse night of sleep was more likely to lead to increased rumination, psychotic-like experiences, and somatic symptoms the following day.

### Subjective sleep duration is not predictive of daytime mental complaints

In contrast to subjective sleep quality, sleep duration on average or daily fluctuations of the duration of sleep were not associated with daytime rumination, psychotic-like experiences, or somatic complaints (Table 3).

### Subjective sleep quality predicts next day functioning regardless of mood fluctuations

Sleep disruption was linked to lower positive mood based on the averages of the study period (between-individuals; b = −0.54, confidence interval [CI]: −0.097 to −0.012, *p* < 0.05), and daily changes in sleep disruption were temporally associated with lower positive mood the next-day (within-individuals; b = −0.06, CI: −0.07 to −0.05, *p* < 0.001). Although negative mood was associated with increased rumination, psychotic-like experiences, and somatic symptoms on both the between- and within-individual levels, the 2-week averages as well as the daily variations in subjective sleep disruption remained significant predictors of the outcome variables beyond the variance explained by negative mood (see Supplementary Table S1). These findings suggest that negative mood did not fully account for the associations of sleep disruption with rumination, psychotic-like experiences, and somatic symptoms.

### Daytime mental complaints and sleep quality and duration the following night

To investigate bidirectional relationships regarding day-to-day associations between sleep and daytime mental health problems, we also examined the temporal associations between daytime mental health complaints and sleep on the following night. Daily rates of rumination, psychotic-like experiences, or negative mood did not significantly predict subjective sleep disruption and sleep duration the following night. Interestingly, daytime somatic complaints showed a negative association with sleep disruption the following night; that is, more somatic complaints during the day (relative to one’s own average) were followed by better sleep quality the following night. On the other hand, higher average level of somatic complaints reported during the study period was associated with poorer sleep quality as shown by a positive association between somatic complaints. Nevertheless, the associations between somatic complaints and sleep disruption did not remain significant after the statistical correction for multiple comparisons. In addition, older age was associated with poorer sleep quality (on average) during the 2-week assessment period (see Table 4). In sum, whereas sleep disruption predicted more mental complaints the following day, such temporal associations in the other direction were only observed at a trend level in case of within-person changes in somatic complaints, whose increases were somewhat paradoxically associated with improvement of subjective sleep quality.

**Table 4.** Summary of mixed models examining associations between daytime functioning and subsequent (next night’s) sleep quality and duration

Associations between daytime mental health complaints and subsequent sleep quality				
	Sleep disruption		Sleep duration	
Predictors	Estimates	95% CI	Estimates	95% CI
Intercept	0.366**	0.122 to 0.610	8.548***	7.758 to 9.338
Age	0.005*	0.001 to 0.009	−0.028***	−0.042 to −0.015
Gender (male)	−0.073	−0.147 to 0.002	−0.436***	−0.677 to −0.195
PTSD-like symptoms	−0.000	−0.003 to 0.003	0.006	−0.004 to 0.017
Cognitive disorganization	0.008	−0.005 to 0.021	−0.024	−0.066 to 0.017
Psychotic-like experiences (within-person mean)	0.005	−0.005 to 0.016	0.026	−0.008 to 0.059
*Psychotic-like experiences (within-person centered)*	*−0.001*	*−0.005 to 0.004*	*0.010*	*−0.006 to 0.025*
Rumination (within-person mean)	0.003	−0.017 to 0.023	−0.050	−0.113 to 0.014
*Rumination (within-person centered)*	*0.004*	*−0.004 to 0.012*	*−0.013*	*−0.038 to 0.013*
Somatic complaints (within-person mean)	0.019**	0.005 to 0.034	−0.016	−0.062 to 0.030
*Somatic complaints (within-person centered)*	−0.008*	*−0.016 to −0.000*	*0.009*	*−0.015 to 0.034*
Mood (within-person mean)	−0.029	−0.066 to 0.008	0.091	−0.028 to 0.211
*Mood (within-person centered)*	*−0.006*	*−0.020 to 0.007*	*−0.004*	*−0.047 to 0.038*
Autocorrelation (dependent variable the day before, within-person centered)	−0.004	−0.010 to 0.002	−0.052*	−0.102 to −0.001
Random effects				
σ ^2^	0.0928		0.9843	
τ _00_	0.0247		0.2567	
ICC	0.2101		0.2069	
*N*	158		158	
Observations	1606		1605	
Marginal *R*^2^/conditional *R*^2^	0.096/0.286		0.068/0.261	

The day-to-day associations between daytime mental health complaints and next night’s sleep disruption are set in italics. Uncorrected *p*-values were computed with Satterthwaite’s approximation. None of the *p*-values corresponding to daytime mental health complaints remained significant after the statistical correction for multiple comparisons. From the examined measures, daytime somatic complaints predicted poorer sleep quality the following night. σ2, residual variance; τ00, variance of random intercept; τ11, variance of random slope; ρ01, correlation between random intercept and slope; ICC, intraclass correlation; *N*, number of participants. Marginal *R*^2^, variance explained by fixed effects; conditional *R*^2^, variance explained by fixed and random effects.

*p < 0.05; **p < 0.01; ***p < 0.001.

### Carryover effects from day-to-day within variables

In order to account for carryover effects and to verify whether the associations between sleep and the next day’s mental health complaints remain significant regardless of the day-to-day associations between the variables, we included the autoregressive parameter in each model. More specifically, outcome variables were also predicted by their respective values at previous time points (autoregressive parameter). This way, we aimed to rule out that the associations between nighttime sleep and the next day’s complaints were not due to carryover effects of mental health complaints from day-to-day (i.e. mental health complaints [day *N* − 1] >> sleep [day *N* − 1] >> mental complaints [day *N*]). We observed significant positive autocorrelation for psychotic-like experiences, rumination, and somatic complaints, suggesting that within-person increases/decreases in these aspects of mental health problems tend to carry over days. Furthermore, we detected that sleep duration had a significant negative autocorrelation, implicating that participants in the sample tended to catch up with sleep debt and to sleep less when they slept more the night before (Table 4). Importantly, sleep quality predicted the next day’s mental complaints (Table 3 and Figure 4) regardless of these carryover effects.

## Discussion

The aim of our study was to prospectively investigate the associations between subjective sleep quality and psychological health in the context of home confinement due to the COVID-19 pandemic. More specifically, 166 individuals from three different countries (out of the 717 respondents in a cross-sectional survey) filled in daily reports during two consecutive weeks. We focused our analyses on the associations at the between-individual level as well as on the bidirectional temporal links between day-to-day variations of sleep quality and daytime rumination, psychotic-like experiences, and somatic complaints within individuals. Our findings indicate that disrupted sleep during the assessment period was associated with more negative psychological conditions such as increased rumination, more psychotic-like experiences, and somatic complaints resembling the most common symptoms of the coronavirus. Furthermore, day-to-day fluctuations covaried within individuals: a relatively poorer night of sleep predicted increased rumination, psychotic-like experiences, and somatic complaints the following day. This association appeared to be mainly unidirectional, since daytime reports of such mental experiences were not significantly associated with poor sleep quality on the following night.

A growing number of studies have reported an alarming increase in the prevalence of mental health complaints during the COVID-19 pandemic [9, 10, 14, 69, 70]. The cross-sectional findings in our samples are in line with these first cross-sectional observations indicating clinically relevant signs of depression and posttraumatic symptomatology in approximately 20% of our respondents. Likewise, impaired sleep quality exhibited moderate associations with retrospectively assessed psychopathological measures [3, 13, 69], such as depression, posttraumatic symptoms, and cognitive disorganization. Since our aim was to include only healthy participants free from severe psychological conditions in our prospective study, we excluded individuals showing clinically relevant signs of depressive symptoms. Nevertheless, we retained and included in our models the scores of PTSD-like symptoms and cognitive disorganization as proxies of psychopathological conditions that were consistently linked to impaired sleep quality [71–74], negative emotionality, and reduced resilience [52–55]. This way, by controlling the effects of the levels of more general psychopathological traits, we could disentangle the associations of sleep quality with daytime reports that reflect specific mental experiences, such as rumination, psychotic-like experiences, and somatic complaints during the confinement.

To evaluate if the perceived circumstances of the pandemic and the confinement influenced our variables of interest, we examined whether the country-specific and global number of deaths as well as the time spent confined at home were predictive of daily measures of mental health problems. Country-specific daily numbers of deaths related to COVID-19 were predictive of more psychotic-like experiences and somatic complaints during the same day, and poorer sleep quality the following night. As for the worldwide daily number of deaths, a less general pattern was observed. There was a positive link with more somatic complaints (the same day) and longer sleep duration the following night. Beyond the associations with COVID-19-related number of deaths, daily variations in rumination, psychotic-like experiences, and somatic complaints were related to the time spent in confinement. On the one hand, we highlight a general improvement in daytime mental health complaints with days elapsed from the introduction of the confinement, but a quadratic (U-shaped) relationship was also observed, indicating that negative psychological states seem to reappear with prolonged confinement. Although we abstain from drawing conclusions regarding the causal relationship of death rates and confinement days with daytime mental health problems, our results suggest that day-to-day variations of negative mental experiences were not independent of the context of the confinement and the pervasive information about the negative consequences of the COVID-19. Previous studies indicate that social media exposure [75] and “local” COVID-19 death counts [15] during the outbreak are risk factors for mental health problems. Our findings are in line with these reports and extend these observations by evidencing a link between death rates and mental health complaints on a day-to-day basis. Nevertheless, we should note that we did not assess whether our participants were aware of the daily reports of COVID-19 death counts nor did we measure the amount of time individuals spent focusing on the media coverage of the pandemic. Future studies should, therefore, investigate these factors in more detail to provide more insights into the links between media use, perceived threat, and mental complaints. The gradual amelioration of mental health complaints as a function of time elapsed since the introduction of the confinement is in line with a previous study showing a reduction of the negative psychological impact of the pandemic 4 weeks after its initial outbreak [76]; however, it is premature to evaluate the long-lasting psychological impact of the confinement based on these findings. Although the number of our observations was relatively lower at later time points (>60 days after the introduction of the confinement), these data indicate that a further prolongation of the confinement period could be associated with negative psychological outcomes [6].

Our results unambiguously indicate that poor subjective sleep quality during the 2-week assessment period was associated with increased rumination, psychotic-like experiences, and somatic complaints about the most typical symptoms of the coronavirus. That is, individuals who exhibited poor subjective sleep quality on average were also more likely to exhibit negative psychological outcomes during the confinement. The outbreak of the pandemic and the extreme circumstances produced by the confinement seem to have had a profound negative impact on subjective sleep quality, and sleep problems consistently correlated with impaired mental health [3, 13, 16, 77]. Our findings based on the prospective assessment of sleep quality and mental health indices corroborate these previous results as well as the well-established role of sleep in emotional adaptation [21, 24, 78]. Furthermore, our data indicate that sleep disruption was associated with rumination, psychotic-like experiences, and somatic complaints over and above the influence of psychopathological traits (PTSD-like symptoms and cognitive disorganization) as well as inter- and intra-individual variations in daily mood. This suggests that the association of sleep quality with more specific mental experiences during the confinement is not merely attributable to general levels of psychopathology and negative affect. At variance with subjective sleep quality, we did not observe similar links between sleep duration and daytime mental health problems. Whereas sleep deprivation and sleep restriction have been consistently associated with increased negative affect [78], pain sensitivity [79], and psychotic-like phenomena [18, 45], links between individual differences in sleep duration and negative health outcomes have only been reported in much larger samples and specifically in the case of extremely short and long sleep durations [80, 81]. Moreover, studies suggest that home confinement provided more freedom to schedule bed- and wake-up times leading to an increase in the time spent in bed [16, 82]. Time in bed, however, does not necessarily correlate with sleep duration, and, what is more, it may reduce the perceived quality of sleep, especially in those individuals who are vulnerable to sleep problems [25].

Our participants exhibited remarkable intraindividual variability in psychological measures and sleep quality during the 2-week study period, allowing for the analyses of day-to-day associations between sleep and daytime mental experiences. Night-to-night variations in sleep quality predicted mental experiences the upcoming day; that is, worse nights were predictive of increased rumination, psychotic-like experiences, and somatic complaints. Furthermore, the associations between sleep quality and the next day’s mental health did not appear to be confounded by carryover effects of mental health complaints between successive days. That is, although the daily reports of rumination, psychotic-like experiences, and somatic complaints were predicted by the same variables assessed the preceding day, these autocorrelations were independent of the influence of the previous night’s sleep quality.

Although the present study does not allow making inferences about the underlying mechanisms linking sleep and daytime mental health, disrupted sleep might facilitate rumination, PLEs, and somatic complaints through a variety of cognitive and affective processes. For instance, impaired sleep exerts a negative impact on prefrontal and frontoparietal networks underlying inhibition-related functions and cognitive flexibility at the behavioral level [83, 84]. These changes, in turn, may enhance ruminative, worrying tendencies and reduce the chance of considering alternative explanations. Furthermore, experimental evidence suggests that impaired and/or restricted sleep causes deficits in attention, oculomotor control, and sensory gating that could, in turn, cause disorganized thought and unusual perceptual experiences that are characteristic of psychotic-like states [85–87]. In addition, impaired fronto-limbic connectivity due to non-efficient sleep might provide the neural background of increased emotional reactivity to stressful situations, including lowered pain thresholds and somatic symptoms [22, 50].

Noticeably, temporal associations between sleep and daytime mental health complaints were not consistent in the reverse direction. Simply put, worse days were not followed by worse nights. Such associations (i.e. daytime stress leading to sleep disruption the following night) have been reported in some studies [38, 88, 89], but others found only unidirectional links between sleep quality and the next day’s psychopathological outcomes [32, 90, 91]. Although a bidirectional relationship between sleep and daytime mental health seems plausible [78], future studies and meta-analyses should corroborate if associations are present in both directions. Interestingly, days with more somatic complaints were followed by less disrupted sleep the following night, although the association was not significant after the correction for multiple comparisons. Although the interpretation of this finding remains elusive, we may speculate that somatic complaints increase fatigue and the homeostatic pressure for sleep. Since we restricted our analyses to individuals who did not self-report prior or current pathological conditions, or above-threshold levels of self-reported depressive states, we cannot here generalize our findings to high-risk or clinical populations, who arguably might be more vulnerable to the adverse effects of the pandemic on mental health. Future studies are needed to explore the potentially stronger and bidirectional temporal associations between sleep difficulties and mental complaints, in order to improve characterization of the need for sleep interventions in vulnerable populations.

The global atmosphere of anxiety and the unusual circumstances of home confinement after the outbreak of the COVID-19 imposed an overwhelming mental health impact on the population. Intrusive and recurrent thoughts, cognitive disorganization and unusual experiences, and dysfunctional anxiety about contracting the coronavirus were common experiences all over the globe. Our results are based on the daily experiences of participants living in European countries that differ in terms of cultural, economic, and political characteristics. The fact that we collected data collection in three different countries accentuates the robustness of our findings in different contexts, reducing the likelihood that country-specific differences in healthcare systems, media use, governmental reactions, and other third variables might largely influence our main findings.

Our results indicate that restorative sleep could be an important factor to counteract the depletion of cognitive resources required for efficient emotional coping strategies during the challenging days of the confinement. Large-scale clinical trials indicate that digital interventions, such as online cognitive behavioral therapy for insomnia, can effectively improve subjective sleep quality and attenuate mental health complaints [72, 93]. The home confinement highlighted the prominent role of online and/or application-based interventions aiming to improve public health during the pandemic [94], and our results call for further development of sleep quality-promoting strategies to help individuals to better cope with such stressful situations; however, the impact of such strategies in the context of COVID-19 remains to be ascertained.

## Methodological statement

The methods were carried out in accordance with the relevant guidelines and regulations.

## Data Availability

Anonymized data and analysis scripts can be found on the project’s OSF page: https://osf.io/xabwe/?view_only=f319528ac4c54e08bb041c788dcb6339.

## Supplementary Material

zsab029_suppl_Supplementary_Materials

## References

[1] Zhou F, et al Clinical course and risk factors for mortality of adult inpatients with COVID-19 in Wuhan, China: a retrospective cohort study. The Lancet. 2020;395(10229):1054–1062. 10.1016/S0140-6736(20)30566-3 PMC7270627 32171076

[2] World Health Organization. Coronavirus disease 2019 (COVID-19): situation report, 72. 2020.

[3] Rajkumar RP . COVID-19 and mental health: a review of the existing literature. Asian J Psychiatr. 2020;52:102066. 32302935 10.1016/j.ajp.2020.102066 PMC7151415

[4] Dutheil F, et al PTSD as the second tsunami of the SARS-Cov-2 pandemic. Psychol Med. 2020;1–2. doi:10.1017/S0033291720001336. PMC7198460 32326997

[5] Bavel JJV, et al Using social and behavioural science to support COVID-19 pandemic response. Nat Hum Behav. 2020;4(5):460–471. 32355299 10.1038/s41562-020-0884-z

[6] Brooks SK, et al The psychological impact of quarantine and how to reduce it: rapid review of the evidence. Lancet. 2020;395(10227):912–920. 32112714 10.1016/S0140-6736(20)30460-8 PMC7158942

[7] Liu N, et al Prevalence and predictors of PTSS during COVID-19 outbreak in China hardest-hit areas: gender differences matter. Psychiatry Res. 2020;287:112921. 32240896 10.1016/j.psychres.2020.112921 PMC7102622

[8] Lai J, et al Factors associated with mental health outcomes among health care workers exposed to Coronavirus disease 2019. JAMA Netw Open. 2020;3(3):e203976–e203976. 32202646 10.1001/jamanetworkopen.2020.3976 PMC7090843

[9] Kaparounaki CK, et al University students’ mental health amidst the COVID-19 quarantine in Greece. Psychiatry Res. 2020;290:113111. 32450416 10.1016/j.psychres.2020.113111 PMC7236729

[10] Odriozola-González P, et al Psychological effects of the COVID-19 outbreak and lockdown among students and workers of a Spanish university. Psychiatry Res. 2020;290:113108. 32450409 10.1016/j.psychres.2020.113108 PMC7236679

[11] Zhao X, et al Perceived stress and sleep quality among the non-diseased general public in China during the 2019 coronavirus disease: a moderated mediation model. Sleep Med. 2020;77:339–345. 32482485 10.1016/j.sleep.2020.05.021 PMC7240276

[12] Wang C, et al Immediate psychological responses and associated factors during the initial stage of the 2019 coronavirus disease (COVID-19) epidemic among the general population in China. Int J Environ Res Public Health. 2020;17(5):1729. 32155789 10.3390/ijerph17051729 PMC7084952

[13] Casagrande M, et al The enemy who sealed the world: effects quarantine due to the COVID-19 on sleep quality, anxiety, and psychological distress in the Italian population. Sleep Med. 2020;75:12–20. 32853913 10.1016/j.sleep.2020.05.011 PMC7215153

[14] Lin L, et al The immediate impact of the 2019 novel coronavirus (COVID-19) outbreak on subjective sleep status. Sleep Med. 2020;77:348–354. 32593614 10.1016/j.sleep.2020.05.018 PMC7831667

[15] Zhang Y, et al Mental health problems during the COVID-19 pandemics and the mitigation effects of exercise: a longitudinal study of college students in China. Int J Environ Res Public Health. 2020;17(10):3722. 32466163 10.3390/ijerph17103722 PMC7277113

[16] Cellini N, et al Changes in sleep pattern, sense of time and digital media use during COVID-19 lockdown in Italy. J Sleep Res. 2020;29(4):e13074. 32410272 10.1111/jsr.13074 PMC7235482

[17] van Liempt S, et al Impact of impaired sleep on the development of PTSD symptoms in combat veterans: a prospective longitudinal cohort study. Depress Anxiety. 2013;30(5):469–474. 23389990 10.1002/da.22054

[18] Barton J, et al Are sleep disturbances causally linked to the presence and severity of psychotic-like, dissociative and hypomanic experiences in non-clinical populations? A systematic review. Neurosci Biobehav Rev. 2018;89:119–131. 29452128 10.1016/j.neubiorev.2018.02.008

[19] Baglioni C, et al Insomnia as a predictor of depression: a meta-analytic evaluation of longitudinal epidemiological studies. J Affect Disord. 2011;135(1-3):10–19. 21300408 10.1016/j.jad.2011.01.011

[20] Freeman D, et al Sleep disturbance and psychiatric disorders. Lancet Psychiatry. 2020;7(7):628–637. 32563308 10.1016/S2215-0366(20)30136-X

[21] Walker MP, et al Overnight therapy? The role of sleep in emotional brain processing. Psychol Bull. 2009;135(5):731–748. 19702380 10.1037/a0016570 PMC2890316

[22] Simon EB, et al Overanxious and underslept. Nat. Hum. Behav. 2019;4:100–110. 31685950 10.1038/s41562-019-0754-8

[23] Wassing R, et al Restless REM sleep impedes overnight Amygdala adaptation. Curr Biol. 2019;29(14):2351–2358.e4. 31303489 10.1016/j.cub.2019.06.034

[24] Deliens G, et al Sleep and the processing of emotions. Exp Brain Res. 2014;232(5):1403–1414. 24449011 10.1007/s00221-014-3832-1

[25] Altena E, et al Dealing with sleep problems during home confinement due to the COVID-19 outbreak: practical recommendations from a task force of the European CBT-I Academy. J Sleep Res. 2020;29(4):e13052. 32246787 10.1111/jsr.13052

[26] Babkoff H, et al A comparison of prospective and retrospective assessments of sleep. J Clin Epidemiol. 1996;49(4):455–460. 8621997 10.1016/0895-4356(95)00529-3

[27] Sato H, et al Selective bias in retrospective self-reports of negative mood states. Anxiety Stress Coping. 2011;24(4):359–367. 21253957 10.1080/10615806.2010.543132

[28] Schroeder DH, et al Influence of life event stress on physical illness: substantive effects or methodological flaws? J Pers Soc Psychol. 1984;46(4):853–863. 6737196 10.1037//0022-3514.46.4.853

[29] Solhan MB, et al Clinical assessment of affective instability: comparing EMA indices, questionnaire reports, and retrospective recall. Psychol Assess. 2009;21(3):425–436. 19719353 10.1037/a0016869 PMC2864015

[30] Kasanova Z, et al Temporal associations between sleep quality and paranoia across the paranoia continuum: an experience sampling study. J Abnorm Psychol. 2020;129(1):122–130. 31343182 10.1037/abn0000453

[31] Hennig T, et al Sleeping paranoia away? An actigraphy and experience–sampling study with adolescents. Child Psychiatry Hum Dev. 2018;49(1):63–72. 28451897 10.1007/s10578-017-0729-9

[32] Simor P, et al Day-to-day variation of subjective sleep quality and emotional states among healthy university students – a 1-week prospective study. Int J Behav Med. 2015;22(5):625–634. 25622815 10.1007/s12529-015-9464-4

[33] Kramer I, et al Time-lagged moment-to-moment interplay between negative affect and paranoia: new insights in the affective pathway to psychosis. Schizophr Bull. 2014;40(2):278–286. 23407984 10.1093/schbul/sbs194 PMC3932075

[34] Fleeson W . Situation-based contingencies underlying trait-content manifestation in behavior. J Pers. 2007;75(4):825–861. 17576360 10.1111/j.1467-6494.2007.00458.x

[35] Nolen-Hoeksema S, et al Rethinking rumination. Perspect Psychol Sci. 2008;3(5):400–424. 26158958 10.1111/j.1745-6924.2008.00088.x

[36] Nolen-Hoeksema S, et al A heuristic for developing transdiagnostic models of psychopathology: explaining multifinality and divergent trajectories. Perspect Psychol Sci. 2011;6(6):589–609. 26168379 10.1177/1745691611419672

[37] Kocsel N, et al The association between perseverative cognition and resting heart rate variability: a focus on state ruminative thoughts. Biol Psychol. 2019;145:124–133. 31051207 10.1016/j.biopsycho.2019.04.004

[38] Pillai V, et al A seven day actigraphy-based study of rumination and sleep disturbance among young adults with depressive symptoms. J Psychosom Res. 2014;77(1):70–75. 24913345 10.1016/j.jpsychores.2014.05.004

[39] Vanderhasselt MA, et al Co-variation between stressful events and rumination predicts depressive symptoms: an eighteen months prospective design in undergraduates. Behav Res Ther. 2016;87:128–133. 27665414 10.1016/j.brat.2016.09.003

[40] Freeman D . Persecutory delusions: a cognitive perspective on understanding and treatment. Lancet Psychiatry. 2016;3(7):685–692. 27371990 10.1016/S2215-0366(16)00066-3

[41] van Os J, et al A systematic review and meta-analysis of the psychosis continuum: evidence for a psychosis proneness–persistence–impairment model of psychotic disorder. Psychol Med. 2009;39(2):179–195. 18606047 10.1017/S0033291708003814

[42] Cristóbal-Narváez P, et al The role of stress-regulation genes in moderating the association of stress and daily-life psychotic experiences. Acta Psychiatr Scand. 2017;136(4):389–399. 28865405 10.1111/acps.12789 PMC5697578

[43] Simor P, et al Poor sleep quality predicts psychotic-like symptoms: an experience sampling study in young adults with schizotypal traits. Acta Psychiatr Scand. 2019;140(2):135–146. 31250426 10.1111/acps.13064

[44] Collip D, et al; G.R.O.U.P. Daily cortisol, stress reactivity and psychotic experiences in individuals at above average genetic risk for psychosis. Psychol Med. 2011;41(11):2305–2315. 21733219 10.1017/S0033291711000602

[45] Reeve S, et al Disrupting sleep: the effects of sleep loss on psychotic experiences tested in an experimental study with mediation analysis. Schizophr Bull. 2018;44(3):662–671. 28981834 10.1093/schbul/sbx103 PMC5890488

[46] Reininghaus UA, et al Unemployment, social isolation, achievement–expectation mismatch and psychosis: findings from the AESOP study. Soc Psychiatry Psychiatr Epidemiol. 2008;43(9):743–751. 18491023 10.1007/s00127-008-0359-4

[47] Rekhi G, et al Impact of distress related to attenuated psychotic symptoms in individuals at ultra high risk of psychosis: findings from the longitudinal youth at risk study. Early Interv Psychiatry. 2019;13(1):73–78. 28560723 10.1111/eip.12451

[48] Lee SA, et al Mental health characteristics associated with dysfunctional coronavirus anxiety. Psychol Med. 2020;1–2. 10.1017/S003329172000121X PMC7184195 32297852

[49] Kroenke K, et al The PHQ-15: validity of a new measure for evaluating the severity of somatic symptoms. Psychosom Med. 2002;64(2):258–266. 11914441 10.1097/00006842-200203000-00008

[50] Finan PH, et al The association of sleep and pain: an update and a path forward. J Pain. 2013;14(12):1539–1552. 24290442 10.1016/j.jpain.2013.08.007 PMC4046588

[51] Buchanan DT, et al Sleep measures predict next-day symptoms in women with irritable bowel syndrome. J Clin Sleep Med.. 2014;10(09):1003–1009. 25142761 10.5664/jcsm.4038 PMC4153105

[52] DiCorcia JA, et al Quotidian resilience: exploring mechanisms that drive resilience from a perspective of everyday stress and coping. Neurosci Biobehav Rev. 2011;35(7):1593–1602. 21513731 10.1016/j.neubiorev.2011.04.008

[53] CICCHETTI D . Resilience under conditions of extreme stress: a multilevel perspective. World Psychiatry. 2010;9(3):145–154. 20975856 10.1002/j.2051-5545.2010.tb00297.x PMC2948722

[54] Polner B, et al The network structure of schizotypy in the general population. Eur Arch Psychiatry Clin Neurosci. 2019. doi:10.1007/s00406-019-01078-x. PMC8119252 31646383

[55] Grant P, et al Schizotypy, social stress and the emergence of psychotic-like states – a case for benign schizotypy? Schizophr Res. 2020;216:435–442. 31796309 10.1016/j.schres.2019.10.052

[56] Buysse DJ, et al The Pittsburgh Sleep Quality Index: a new instrument for psychiatric practice and research. Psychiatry Res. 1989;28(2):193–213. 2748771 10.1016/0165-1781(89)90047-4

[57] Beck AT, et al Screening depressed patients in family practice. A rapid technic. Postgrad Med. 1972;52(6):81–85. 4635613 10.1080/00325481.1972.11713319

[58] Mason O, et al Short scales for measuring schizotypy. Schizophr Res. 2005;78(2-3):293–296. 16054803 10.1016/j.schres.2005.06.020

[59] Blevins CA, et al The posttraumatic stress disorder checklist for DSM-5 (PCL-5): development and initial psychometric evaluation. J Trauma Stress. 2015;28(6):489–498. 26606250 10.1002/jts.22059

[60] Hale T, et al Oxford COVID-19 Government Response Tracker, Blavatnik School of Government. https://covidtracker.bsg.ox.ac.uk/.

[61] McCubbin JA, et al Subclinical posttraumatic stress disorder symptoms: relationships with blood pressure, hostility, and sleep. Cardiovasc Psychiatry Neurol. 2016;2016:4720941. 27403340 10.1155/2016/4720941 PMC4925987

[62] Meijman TF, et al. The Evaluation of the Groningen Sleep Quality Scale. Groningen: Heymans Bulletin (HB 88-13-EX); 1988: 2006.

[63] Simor P, et al A questionnaire based study of subjective sleep quality: the psychometric evaluation of the Hungarian version of the Groningen Sleep Quality Scale. Mentalhig Pszichoszom. 2009;10(3):249–261.

[64] Mason OJ, et al The psychotomimetic states inventory (PSI): measuring psychotic-type experiences from ketamine and cannabis. Schizophr Res. 2008;103(1-3):138–142. 18387788 10.1016/j.schres.2008.02.020

[65] Bates D, et al Fitting linear mixed-effects models using lme4. J Stat Softw. 2015;67(1):1–48.

[66] Wang LP, et al On disaggregating between-person and within-person effects with longitudinal data using multilevel models. Psychol Methods. 2015;20(1):63–83. 25822206 10.1037/met0000030

[67] Benjamini Y, et al Controlling the false discovery rate: a practical and powerful approach to multiple testing. J R Stat Soc Series B Stat Methodol. 1995;57(1):289–300.

[68] Furlanetto LM, et al The validity of the Beck Depression Inventory-short form as a screening and diagnostic instrument for moderate and severe depression in medical inpatients. J Affect Disord. 2005;86(1):87–91. 15820275 10.1016/j.jad.2004.12.011

[69] Huang Y, et al Generalized anxiety disorder, depressive symptoms and sleep quality during COVID-19 outbreak in China: a web-based cross-sectional survey. Psychiatry Res. 2020;288:112954. 32325383 10.1016/j.psychres.2020.112954 PMC7152913

[70] Kwong ASF, et al Mental health during the COVID-19 pandemic in two longitudinal UK population cohorts. medRxiv, doi:10.1101/2020.06.16.20133116, 2020, preprint: not peer reviewed PMC7844173 33228822

[71] Tempesta D, et al Long-term impact of earthquakes on sleep quality. PLoS One. 2013;8(2):e55936. 23418478 10.1371/journal.pone.0055936 PMC3572187

[72] Charuvastra A, et al Safe enough to sleep: sleep disruptions associated with trauma, posttraumatic stress, and anxiety in children and adolescents. Child Adolesc Psychiatr Clin N Am. 2009;18(4):877–891. 19836694 10.1016/j.chc.2009.04.002

[73] Ross RJ, et al Sleep disturbance as the hallmark of posttraumatic stress disorder. Am J Psychiatry. 1989;146(6):697–707. 2658624 10.1176/ajp.146.6.697

[74] Reeve S, et al The role of sleep dysfunction in the occurrence of delusions and hallucinations: a systematic review. Clin Psychol Rev. 2015;42:96–115. 26407540 10.1016/j.cpr.2015.09.001 PMC4786636

[75] Gao J, et al Mental health problems and social media exposure during COVID-19 outbreak. PLoS One. 2020;15(4):e0231924. 32298385 10.1371/journal.pone.0231924 PMC7162477

[76] Wang C, et al A longitudinal study on the mental health of general population during the COVID-19 epidemic in China. Brain Behav Immun. 2020;87:40–48. 32298802 10.1016/j.bbi.2020.04.028 PMC7153528

[77] Li D-J, et al Covid-19-related factors associated with sleep disturbance and suicidal thoughts among the Taiwanese public: a Facebook survey. Int J Environ Res Public Health. 2020;17(12):4479. 32580433 10.3390/ijerph17124479 PMC7345275

[78] Ben Simon E, et al Sleep loss and the socio-emotional brain. Trends Cogn Sci. 2020;24(6):435–450. 32299657 10.1016/j.tics.2020.02.003

[79] Schrimpf M, et al The effect of sleep deprivation on pain perception in healthy subjects: a meta-analysis. Sleep Med. 2015;16(11):1313–1320. 26498229 10.1016/j.sleep.2015.07.022

[80] Cappuccio FP, et al Sleep duration and all-cause mortality: a systematic review and meta-analysis of prospective studies. Sleep. 2010;33(5):585–592. 20469800 10.1093/sleep/33.5.585 PMC2864873

[81] Itani O, et al Short sleep duration and health outcomes: a systematic review, meta-analysis, and meta-regression. Sleep Med. 2017;32:246–256. 27743803 10.1016/j.sleep.2016.08.006

[82] Marelli S, et al Impact of COVID-19 lockdown on sleep quality in university students and administration staff. J. Neurol. 2021;268(1):8–15. 32654065 10.1007/s00415-020-10056-6 PMC7353829

[83] Killgore WDS . Effects of sleep deprivation on cognition. Prog Brain Res.. 2010;185:105–129. 21075236 10.1016/B978-0-444-53702-7.00007-5

[84] Nishida M, et al Sleep complaints are associated with reduced left prefrontal activation during a verbal fluency task in patients with major depression: a multi-channel near-infrared spectroscopy study. J Affect Disord. 2017;207:102–109. 27721182 10.1016/j.jad.2016.09.028

[85] Andrillon T, et al Does the mind wander when the brain takes a break? Local sleep in wakefulness, attentional lapses and mind-wandering. Front Neurosci. 2019;13:949. 31572112 10.3389/fnins.2019.00949 PMC6753166

[86] Petrovsky N, et al Sleep deprivation disrupts prepulse inhibition and induces psychosis-like symptoms in healthy humans. J Neurosci. 2014;34(27):9134–9140. 24990933 10.1523/JNEUROSCI.0904-14.2014 PMC6608255

[87] Meyhöfer I, et al Combining two model systems of psychosis: the effects of schizotypy and sleep deprivation on oculomotor control and psychotomimetic states. Psychophysiology. 2017;54(11):1755–1769. 28714081 10.1111/psyp.12917

[88] AAkerstedt T, et al Predicting sleep quality from stress and prior sleep–a study of day-to-day covariation across six weeks. Sleep Med. 2012;13(6):674–679. 22621983 10.1016/j.sleep.2011.12.013

[89] Doane LD, et al Associations among sleep, daily experiences, and loneliness in adolescence: evidence of moderating and bidirectional pathways. J Adolesc. 2014;37(2):145–154. 24439620 10.1016/j.adolescence.2013.11.009

[90] Bouwmans MEJ, et al Sleep quality predicts positive and negative affect but not vice versa. An electronic diary study in depressed and healthy individuals. J Affect Disord. 2017;207:260–267. 27736737 10.1016/j.jad.2016.09.046

[91] Galambos NL, et al Who sleeps best? Longitudinal patterns and covariates of change in sleep quantity, quality, and timing across four university years. Behav Sleep Med. 2013;11(1):8–22. 23347113 10.1080/15402002.2011.596234

[92] Espie CA, et al Effect of digital cognitive behavioral therapy for Insomnia on health, psychological well-being, and sleep-related quality of life: a randomized clinical trial. JAMA Psychiatry. 2019;76(1):21–30. 30264137 10.1001/jamapsychiatry.2018.2745 PMC6583463

[93] Freeman D, et al The effects of improving sleep on mental health (OASIS): a randomised controlled trial with mediation analysis. Lancet Psychiatry. 2017;4(10):749–758. 28888927 10.1016/S2215-0366(17)30328-0 PMC5614772

[94] Zhou X, et al The role of telehealth in reducing the mental health burden from COVID-19. Telemed J E Health. 2020;26(4):377–379. 32202977 10.1089/tmj.2020.0068

