# Supplementary material for: Home confinement during the COVID-19: day-to-day associations of sleep quality with rumination, psychotic-like experiences, and somatic symptoms
Source: Sleep. 2021 Feb 10;44(7):zsab029. doi: 10.1093/sleep/zsab029 (PMC7928634; doi:10.1093/sleep/zsab029)
Supplement: zsab029_suppl_Supplementary_Materials [file zsab029_suppl_supplementary_materials.docx]

**Home confinement during the COVID-19: day-to-day associations of sleep quality with rumination, psychotic-like experiences, and somatic symptoms**

- **Supplementary Materials**-

Péter Simor^* 1,2^, Polner B^* 3^, Báthori N^3^,  Sifuentes-Ortega R^2^, Van Roy A^2^ Albajara Sáenz A^2^, Luque González A^4^, Benkirane O^2^, Nagy T^1^, Peigneux P^2^.

*1 Institute of Psychology, ELTE, Eötvös Loránd University, Budapest, Hungary.*

*2 UR2NF, Neuropsychology and Functional Neuroimaging Research Unit at CRCN - Center for Research in Cognition and Neurosciences and UNI - ULB Neurosciences Institute, Université Libre de Bruxelles (ULB), Brussels, Belgium.*

*3 Budapest University of Technology and Economics, Department of Cognitive Science*

*4 Universidad Autónoma de Madrid,* Department of Biological and Health Psychology

**Corresponding author:**

Péter Simor, Ph.D.

Institute of Psychology, ELTE Eötvös Loránd University, Budapest, Hungary.

1064 Budapest, Izabella utca 46.

simor.peter@ppk.elte.hu

+36302755968

* The authors contributed equally to this work.

**S1. Cross-sectional comparisons across the three sites, Belgium (N = 227), Spain (N = 283) and Hungary (N = 207).** Age showed the most pronounced difference across the three countries (BE vs ESP: t = -5.41, d = -0.41, p < 0.0001; BE vs HUN t = 6.14, d = 0.71, p < 0.0001; ESP vs HUN t = 11.73, d = 1.11, p < 0.0001). Posttraumatic symptoms (PCL), Cognitive Disorganization (Disorg), and the increase in perceived stress due to the COVID-19 (Stress) exhibited significant differences across countries. Depression (BDI), and Sleep disturbance (Sleep) did not significantly differ across countries. The effect size (Cohen’s d) of the differences marked as significant ranged between 0.3 and 0.47). *** - FDR corrected p <0.001, ** - FDR corrected p < 0.01.

| **Associations between sleep disruption and mental health complaints the following day, controlled for mood** | | | | | | |
| --- | --- | --- | --- | --- | --- | --- |
|  | **Rumination** | | **Psychotic-like experiences** | | **Somatic complaints** | |
| *Predictors* | *Estimates* | *95% CI* | *Estimates* | *95% CI* | *Estimates* | *95% CI* |
| Intercept | 0.621 ^*^ | 0.036 – 1.207 | 0.400 | -0.310 – 1.110 | 0.454 | -0.111 – 1.020 |
| Age | 0.000 | -0.005 – 0.005 | -0.004 | -0.010 – 0.003 | -0.004 | -0.009 – 0.001 |
| Gender (male) | -0.097 ^*^ | -0.186 – -0.008 | -0.036 | -0.145 – 0.072 | -0.112 ^*^ | -0.199 – -0.024 |
| PTSD-like symptoms | 0.007 ^***^ | 0.004 – 0.010 | 0.009 ^***^ | 0.005 – 0.013 | 0.006 ^***^ | 0.003 – 0.009 |
| Cognitive Disorganisation | 0.014 | -0.000 – 0.029 | 0.024 ^**^ | 0.006 – 0.042 | 0.019 ^*^ | 0.004 – 0.033 |
| Sleep disruption (within-person mean) | 0.023 ^*^ | 0.001 – 0.045 | 0.052 ^***^ | 0.026 – 0.079 | 0.054 ^***^ | 0.032 – 0.075 |
| Sleep disruption (within-person centered) | 0.005 | -0.001 – 0.011 | 0.006 ^*^ | 0.001 – 0.012 | 0.005 ^*^ | 0.000 – 0.009 |
| Sleep duration (within-person mean) | -0.025 | -0.084 – 0.034 | -0.004 | -0.076 – 0.067 | -0.004 | -0.061 – 0.053 |
| Sleep duration (within-person centered) | -0.010 | -0.025 – 0.004 | -0.003 | -0.019 – 0.012 | -0.009 | -0.022 – 0.003 |
| Autocorrelation (dependent variable the day before, within-person centered) | 0.016 ^***^ | 0.010 – 0.021 | 0.008 ^***^ | 0.005 – 0.011 | 0.006 ^*^ | 0.001 – 0.012 |
| Positive mood (within-person mean) | -0.053 ^*^ | -0.095 – -0.010 | -0.045 | -0.097 – 0.007 | -0.041 | -0.082 – -0.000 |
| Positive mood (within-person centered) | -0.059 ^***^ | -0.069 – -0.048 | -0.087 ^***^ | -0.098 – -0.076 | -0.051 ^***^ | -0.060 – -0.041 |
| **Random Effects** | | | | | | |
| σ^2^ | 0.0697 | | 0.0757 | | 0.0494 | |
| τ_00_ | 0.0403 _id_ | | 0.0625 _id_ | | 0.0377 _id_ | |
| τ_11_ | 0.0002 _id.gsqs_sum_centered_ | |  | |  | |
| ρ_01_ | 0.0614 _id_ | |  | |  | |
| ICC | 0.3750 | | 0.4523 | | 0.4325 | |
| N | 165 _id_ | | 165 _id_ | | 157 _id_ | |
| Observations | 1524 | | 1524 | | 1454 | |
| Marginal R^2^ / Conditional R^2^ | 0.216 / 0.510 | | 0.308 / 0.621 | | 0.320 / 0.614 | |
| ** p<0.05   ** p<0.01   *** p<0.001* | | | | | | |

**Table S1.** Summary of mixed models examining associations between sleep and subsequent daytime functioning, controlled for mood. σ2: residual variance; τ00: variance of random intercept; τ11: variance of random slope; ρ01: correlation between random intercept and slope; ICC: intraclass correlation; N: number of participants. Marginal R^2^: variance explained by fixed effects;  Conditional R^2^: variance explained by fixed and random effects. P-values were computed with Satterthwaite's approximation.

**Models including Country as a fixed factor**

| **Table S2. Associations between sleep disruption and subsequent daytime mental health complaints** | | | | | | |
| --- | --- | --- | --- | --- | --- | --- |
|  | **Rumination** | | **Psychotic-like experiences** | | **Somatic complaints** | |
| *Predictors* | *Estimates* | *95% CI* | *Estimates* | *95% CI* | *Estimates* | *95% CI* |
| Intercept | 0.095 | -0.089 – 0.278 | 0.158 | -0.056 – 0.373 | 0.343 ^***^ | 0.170 – 0.516 |
| Age | 0.002 | -0.004 – 0.007 | -0.002 | -0.008 – 0.004 | -0.006 ^*^ | -0.011 – -0.001 |
| Gender (male) | -0.073 | -0.160 – 0.015 | -0.016 | -0.117 – 0.086 | -0.110 ^**^ | -0.192 – -0.029 |
| PTSD-like symptoms | 0.007 ^***^ | 0.003 – 0.010 | 0.009 ^***^ | 0.005 – 0.013 | 0.006 ^***^ | 0.003 – 0.009 |
| Cognitive Disorganisation | 0.020 ^**^ | 0.005 – 0.035 | 0.027 ^**^ | 0.010 – 0.044 | 0.022 ^**^ | 0.009 – 0.036 |
| Sleep disruption (within-person mean) | 0.029 ^*^ | 0.007 – 0.052 | 0.050 ^***^ | 0.024 – 0.076 | 0.050 ^***^ | 0.029 – 0.070 |
| Sleep disruption (within-person centered) | 0.014 ^**^ | 0.005 – 0.023 | 0.020 ^***^ | 0.011 – 0.029 | 0.013 ^**^ | 0.005 – 0.021 |
| Country (Spain) | -0.028 | -0.130 – 0.074 | -0.134 ^*^ | -0.254 – -0.014 | -0.032 | -0.128 – 0.064 |
| Country (Hungary) | 0.004 | -0.079 – 0.087 | -0.070 | -0.168 – 0.027 | -0.127 ^**^ | -0.203 – -0.052 |
| Autocorrelation (dependent variable the day before, within-person centered) | 0.016 ^***^ | 0.010 – 0.022 | 0.007 ^***^ | 0.004 – 0.011 | 0.006 ^*^ | 0.000 – 0.011 |
| Sleep disruption (within-person centered) x Spain | -0.018 | -0.036 – 0.000 | -0.016 | -0.035 – 0.003 | -0.015 | -0.032 – 0.003 |
| Sleep disruption (within-person centered) x Hungary | -0.006 | -0.018 – 0.007 | -0.016 ^*^ | -0.029 – -0.003 | -0.005 | -0.017 – 0.006 |
| Observations | 1524 | | 1524 | | 1454 | |
| Marginal R^2^ / Conditional R^2^ | 0.164 / 0.470 | | 0.256 / 0.567 | | 0.311 / 0.596 | |
| ** p<0.05   ** p<0.01   *** p<0.001* | | | | | | |

| **Table S3. Associations between sleep duration and subsequent daytime mental health complaints** | | | | | | |
| --- | --- | --- | --- | --- | --- | --- |
|  | **Rumination** | | **Psychotic-like experiences** | | **Somatic complaints** | |
| *Predictors* | *Estimates* | *95% CI* | *Estimates* | *95% CI* | *Estimates* | *95% CI* |
| Intercept | 0.585 | -0.012 – 1.182 | 0.724 | 0.004 – 1.445 | 0.940 ^**^ | 0.366 – 1.514 |
| Age | 0.001 | -0.005 – 0.007 | -0.002 | -0.009 – 0.005 | -0.007 ^*^ | -0.012 – -0.001 |
| Gender (male) | -0.113 ^*^ | -0.204 – -0.023 | -0.073 | -0.182 – 0.037 | -0.177 ^***^ | -0.264 – -0.089 |
| PTSD-like symptoms | 0.008 ^***^ | 0.005 – 0.011 | 0.011 ^***^ | 0.007 – 0.015 | 0.007 ^***^ | 0.004 – 0.011 |
| Cognitive Disorganisation | 0.022 ^**^ | 0.007 – 0.037 | 0.032 ^***^ | 0.014 – 0.050 | 0.027 ^***^ | 0.012 – 0.041 |
| Sleep duration (within-person mean) | -0.047 | -0.109 – 0.015 | -0.053 | -0.128 – 0.021 | -0.054 | -0.113 – 0.005 |
| Sleep duration (within-person centered) | -0.009 | -0.034 – 0.016 | -0.012 | -0.040 – 0.016 | 0.004 | -0.020 – 0.029 |
| Country (Spain) | -0.049 | -0.153 – 0.055 | -0.165 ^*^ | -0.291 – -0.039 | -0.054 | -0.156 – 0.049 |
| Country (Hungary) | -0.031 | -0.114 – 0.052 | -0.122 ^*^ | -0.223 – -0.022 | -0.182 ^***^ | -0.261 – -0.103 |
| Autocorrelation (dependent variable the day before, within-person centered) | 0.016 ^***^ | 0.010 – 0.022 | 0.007 ^***^ | 0.004 – 0.011 | 0.006 | -0.000 – 0.011 |
| Sleep duration (within-person centered) x Spain | 0.018 | -0.034 – 0.070 | 0.017 | -0.040 – 0.073 | -0.017 | -0.066 – 0.031 |
| Sleep duration (within-person centered) x Hungary | -0.015 | -0.047 – 0.017 | 0.002 | -0.033 – 0.038 | -0.026 | -0.057 – 0.006 |
| Observations | 1524 | | 1524 | | 1454 | |
| Marginal R^2^ / Conditional R^2^ | 0.152 / 0.458 | | 0.224 / 0.559 | | 0.274 / 0.590 | |
| ** p<0.05   ** p<0.01   *** p<0.001* | | | | | | |

| **Table S4. Associations between daytime mental health complaints and subsequent sleep** | | | | |
| --- | --- | --- | --- | --- |
|  | **Sleep disruption** | | **Sleep duration** | |
| *Predictors* | *Estimates* | *95% CI* | *Estimates* | *95% CI* |
| Intercept | 0.431 ^***^ | 0.182 – 0.679 | 8.680 ^***^ | 7.870 – 9.490 |
| Age | 0.003 | -0.002 – 0.008 | -0.030 ^***^ | -0.045 – -0.016 |
| Gender (male) | -0.079 ^*^ | -0.154 – -0.005 | -0.436 ^***^ | -0.678 – -0.194 |
| PTSD-like symptoms | -0.000 | -0.003 – 0.003 | 0.007 | -0.004 – 0.017 |
| Cognitive Disorganisation | 0.009 | -0.004 – 0.021 | -0.025 | -0.067 – 0.016 |
| Psychotic-like experiences (within-person mean) | 0.005 | -0.006 – 0.015 | 0.021 | -0.014 – 0.055 |
| Psychotic-like experiences (within-person centered) | -0.001 | -0.005 – 0.004 | 0.009 | -0.006 – 0.025 |
| Rumination (within-person mean) | 0.007 | -0.013 – 0.027 | -0.039 | -0.104 – 0.026 |
| Rumination (within-person centered) | 0.004 | -0.004 – 0.012 | -0.013 | -0.038 – 0.013 |
| Somatic complaints (within-person mean) | 0.015 ^*^ | 0.001 – 0.030 | -0.022 | -0.069 – 0.026 |
| Somatic complaints (within-person centered) | -0.008 ^*^ | -0.016 – -0.001 | 0.009 | -0.015 – 0.034 |
| Mood (within-person mean) | -0.025 | -0.062 – 0.012 | 0.099 | -0.021 – 0.218 |
| Mood (within-person centered) | -0.006 | -0.020 – 0.007 | -0.004 | -0.047 – 0.038 |
| Country (Spain) | 0.017 | -0.070 – 0.104 | -0.112 | -0.395 – 0.170 |
| Country (Hungary) | -0.070 | -0.140 – 0.001 | -0.182 | -0.412 – 0.048 |
| Autocorrelation (dependent variable the day before, within-person centered) | -0.004 | -0.010 – 0.002 | -0.051 ^*^ | -0.101 – -0.001 |
| Observations | 1606 | | 1605 | |
| Marginal R^2^ / Conditional R^2^ | 0.104 / 0.289 | | 0.072 / 0.263 | |
| ** p<0.05   ** p<0.01   *** p<0.001* | | | | |
